# Caution on corticosteroids for preterm delivery: learning from missteps

**DOI:** 10.9745/GHSP-D-14-00197

**Published:** 2014-12-02

**Authors:** Stephen Hodgins

**Affiliations:** a Global Health: Science and Practice, Associate Editor for Maternal and Child Health.

## Abstract

An important new study in lower-level health facilities in low- and middle-income countries found an increased risk of neonatal deaths with corticosteroid use in pregnant women with imminent preterm birth, in contrast with the positive results previously found in high-income countries. The surprising finding demonstrates that *context matters*. The increase appears largely due to steroids administered in cases that were *not* actually preterm, probably due to inaccurate pregnancy dating and challenges with diagnostic capacity. Promoting public health often requires decisions based on less-than-perfect evidence, but we must be vigilant about gathering and assessing new evidence and ready to change strategies.

Globally, preterm birth, along with pneumonia, is the leading cause of death among children under 5.^1^ The use of corticosteroids for cases of imminent preterm birth has significantly reduced the burden of such deaths in high-income countries. Numerous studies have confirmed important effects, particularly in reducing risk of respiratory distress syndrome (RDS) through its action on production of lung surfactant. A 2006 Cochrane review found a 31% reduction in risk of neonatal death, across 21 studies conducted in middle- and high-income countries.^2^ On the strength of this substantial evidence, corticosteroids for this indication have been included among the interventions that are the focus of the UN Commission on Life-Saving Commodities for Women and Children,^3^ adding to the momentum to expand use of this intervention in lower-income settings with less sophisticated health services.

## NEW EVIDENCE

As part of this effort to extend the use of steroids, a very large multicountry, cluster-randomized trial was conducted recently, testing a strategy to push such services more peripherally to the community, health center, and district hospital levels. Findings were published by Althabe and colleagues this year on October 15 in the *Lancet*.^4^ The results come as something of a shock.

In this 6-country study (with sites in Argentina, Guatemala, India, Kenya, Pakistan, and Zambia), 99,742 pregnant women were enrolled. In the intervention arm, 6,109 women received steroids (dexamethasone). The main outcome tracked was neonatal mortality, measured overall and among those in the lowest 5% by birth weight (as a proxy for preterm births, which couldn't be reliably determined for the whole cohort). Overall neonatal mortality was 12% higher in the intervention arm (relative risk [RR] = 1.12, 95% confidence interval [CI] = 1.02–1.22), and the stillbirth rate was similarly elevated. Among those in the lowest 5% by birth weight, mortality was the same in intervention and control arms (RR = 0.96, 95% CI = 0.87–1.06). Risk of maternal infection was also increased; among those with births in the lowest 5% by birth weight, infection was reported in 10% of the women in the intervention group vs. 6% in the control group (odds ratio [OR] = 1.67, 95% CI = 1.33–2.09).

## NET HARM? HOW COULD THAT BE?

The elevated risk in overall newborn mortality appears to have been largely due to administration of dexamethasone in cases that were actually *not* preterm at the time of delivery. There had been previous hints of safety issues for cases delivering at or near term; the Cochrane review found elevated mortality for steroid-exposed cases going to term or near-term compared with controls, with borderline statistical significance.^2^ Results of the new study confirm this elevated risk. There had also been suggestive evidence in the Cochrane review of elevated risk of *maternal* infection, which was confirmed by the new study.

Why the detrimental effects among those delivering later? One can only speculate. But corticosteroids directly or indirectly influence virtually all major systems in the body. They have effects on: stress response; metabolism; renal function; cardiovascular and endocrine functions; muscle and bone; central nervous system functions; eyes and skin; gut function; and—*perhaps of most relevance to this situation*—growth and development and inflammatory and immune response (with consequences for vulnerability to infection).^5^ It's not hard to imagine that some combination of such effects from steroid exposure in late pregnancy—when risk of RDS is much reduced—could result in net harm.

## IS THERE NO BENEFIT TO PRETERM NEWBORNS AFTER ALL?

The results of the new study did not show net benefit among those in the bottom 5% by birth weight. But this weight category is a mixed bag, comprising virtually all those born at <34 weeks gestation (ie, those who were actually preterm) but also a significant number of growth-restricted term or near-term newborns. The lack of a measured beneficial effect among those in the bottom 5%, taken as a group, could mask a situation in which there was a real benefit among those actually preterm at birth, but this was offset by an elevated risk among those born at or near term. Furthermore, even among those genuinely preterm, there may have been an attenuation of a real effect: since the study sites generally lacked the capacity to adequately manage complications in preterm newborns, those born very premature (say, <28 weeks gestation) may not have experienced lower mortality even if their risk of RDS was reduced with the dexamethasone treatment.

The lack of measured benefit among very low birth weight newborns may reflect real benefit among those genuinely preterm, offset by an elevated risk among those born at or near term.

## WHAT HAVE WE LEARNED?

Our strategic stance had been: *Push this intervention further out; save more newborn lives*. In hindsight, although the potential benefits of corticosteroids are substantial, this experience argues for more consideration of safety, given the particular vulnerability of fetal life and the potent and manifold effects of these drugs.

Possible risks had not received much attention in the published literature. In none of the 21 trials summarized in the Cochrane review was there disaggregated analysis of steroid-exposed cases that went on to deliver at or near term. Only by obtaining additional data not included in the original paper by Liggins^7^ (one of the trials summarized in the review) did the authors of the Cochrane review have sufficient data to do this secondary analysis. But this was one sub-analysis of many dozens done in the review; in the face of an overwhelmingly positive picture from the analysis as a whole, this potentially worrying suggestion of adverse effects was not flagged for special attention in the discussion or conclusions of the review. We overlooked a small grey cloud in an otherwise clear blue sky. Following publication of the protocol for the Althabe study, there was no published objection to the design on the basis of safety. Only in spring 2014, by which time study enrollment was closed, was a note of alarm first rung in the published literature.^6^

These findings serve as a reminder that “effect sizes” cannot be considered context-free. In this case, they are a function of the interaction between *agent* (dexamethasone), *host* (with its responses driven in part, for example, by nutritional status and infectious and parasitic exposures), and the service delivery *environment* (notably, capacity to manage complications).

In public health, all decisions, even decisions to do nothing, have consequences. As a general rule, we have to make decisions based on less-than-perfect evidence. Then, as we proceed, we need to keep our critical faculties alert, continue to gather evidence, and, when required, be ready to revisit previously drawn conclusions in the light of new evidence. With use of antenatal corticosteroids, we have reached such a juncture.

## WHAT NOW?

At this point, clearly, the global newborn community will need to sharply adjust its stance on steroid use for management of pregnant women facing imminent preterm delivery. The World Health Organization is expected, in the near future, to issue new, more explicitly conservative guidelines.

The imperative of extending the benefits of this efficacious intervention to lower-resource settings remains. But it is now evident that corticosteroid use needs to be restricted to clinical settings where it can be ensured the drug is given only for cases that will deliver preterm (<34 weeks gestation). This is not necessarily an easy standard to meet. Accurate dating requires early antenatal contact and, ideally, obstetrical ultrasound. Diagnosis of imminent preterm delivery also requires sound diagnostic capability. Furthermore, to derive full benefit from this intervention, close monitoring and appropriate care needs to be reliably available to manage common life-threatening complications of prematurity. This requirement, also, is not necessarily easy to meet. Even tertiary care centers categorized as providing level-2 newborn care may be stretched quite thin and may struggle to meet such requirements.

Corticosteroid use should be restricted to clinical settings where it can be ensured the drug is given only for preterm deliveries.

It is inevitable that new evidence will come along from time to time that calls into question previous decisions or strategies, as it has in this case. As public health professionals, we need to be ready to learn from our missteps, adjust our perspectives, and change strategy in the face of such evidence.
